# Work-related COPD after years of occupational exposure

**DOI:** 10.1186/s40557-015-0056-1

**Published:** 2015-02-19

**Authors:** YounMo Cho, JongIn Lee, Min Choi, WonSeon Choi, Jun-Pyo Myong, Hyoung-Ryoul Kim, Jung-Wan Koo

**Affiliations:** Department of Occupational & Environmental Medicine, Seoul St. Mary’s Hospital, Seoul, Republic of Korea; Department of Occupational & Environmental Medicine, Seoul St. Mary’s Hospital, College of Medicine, The Catholic University of Korea, 222 Banpo-Daero Seocho-gu, Seoul, 137-701 Republic of Korea; Center for Occupational and Environmental Medicine, The Catholic University of Korea, Seoul St. Mary’s Hospital, Seoul, Republic of Korea; Department of Occupational & Environmental Medicine, Korea Medical Institute, Suwon, Republic of Korea

**Keywords:** COPD, Occupational exposure, Fuel oils, Silica

## Abstract

**Background:**

Cigarette smoking is known as the most important risk factor of chronic obstructive pulmonary disease (COPD). However, occupational exposure to other substances can result in COPD.

**Case report:**

A 76-year-old man with occupational exposures to mixtures of silica dust, gas, and fumes for 10 years and with a 25 pack-year smoking history was diagnosed with COPD. His computed tomogram scan revealed some hyperinflation with emphysematous change in both upper lobes. In the pulmonary function tests, his post-bronchodilator forced vital capacity (FVC), forced expiratory volume in one second (FEV_1_), and FEV_1_/FVC% were 2.20 L (67% of the predicted value), 1.12 L (52% of the predicted value), and 51%, respectively, indicating moderate COPD. This case of COPD was confirmed as a work-related disease by the Occupational Lung Disease Research Institute in Korea Workers’ Compensation & Welfare Service.

**Conclusion:**

Exposure to various substances such as silica dust, gas, and fumes from furnace and boiler installation was likely the cause of COPD in this patient. Thus, occupational exposure should be considered an important risk factor of COPD.

## Background

Chronic obstructive pulmonary disease (COPD) is characterized by the irreversible and progressive small airway obstruction that develops when a patient inhales harmful gas or particles [1]. The most important risk factor of developing COPD is cigarette smoking; however, occupational exposure, exposure to air pollutions, and respiratory infections can also be attributed to developing COPD [2,3]. In addition, the dusts from coal, stone quarries, wood, cereals and agricultural work, animal stables, textiles, and paper production that can arise in occupational environments have been regulated by the International Labor Organization and considered possible as contributors to COPD [4]. A previous review calculated the population attributable risk for COPD and reported that 15% of the diagnosed COPD was related to one’s occupation [5,6]. However, cases of occupational COPD might have been underreported. Using data from 1998–2007 by the Occupational Safety and Health Research Institute of the Korea Occupational Safety & Health Agency, Lee et al. [7] reported that only four cases out of 13 cases of occupational COPD were approved for their work relatedness. On July 1, 2013, COPD (related to coal dust, silica dust, and other occupational exposures) was listed as a compensable occupational disease under the Enforcement Decree of the Industrial Accident Compensation Insurance. Therefore, an evaluation of the relation between COPD incidence and exposure to various hazardous materials (cigarette smoke, silica dust, coal dust, gases, fumes, etc.) in the work environment has become a key issue.

Recently, we diagnosed a patient with COPD and aimed to evaluate the incidence of disease with the longtime exposure to various toxins including silica dust, gases, and fumes while working at a wheel manufacturing factory. This patient presented with a high occupational hazard and was admitted after an industrial accident. We report this case to focus on the fact that occupational exposure is one of the main risk factors of developing COPD along with a history of cigarette smoking.

## Case presentation

A 76-year-old man presented with a 7-year history of dyspnea on exertion. He had received intermittent medical treatment that only relieved his symptoms. The patient had smoke for 25 pack-years and quit at the age of 66. According to his medical history, there was no evidence of tuberculosis.

Table 1 presents the patient’s occupational history. He reported 10 years of occupational exposure to fumes from a heat-treatment procedure and silica dust. He had worked at a wheel manufacturing company from Feb 7, 1965 to Mar 7, 1975.

**Table 1:** **The patient’s occupational history**

**Job or Worksite**	**Duration (years)**	**Task**	**Possible Exposure**	**Exposure levels in the literature**
Wheel manufacturing company	1965–1975 (10)	Transported the casting crane to the heating furnace and controlled the temperature of the furnace	Bunker-C oil (gas and/or fumes)	PAHs 11,056.61 ng/Sm^3^ [23]
			Silica dust (quartz)	Personal sampling 4.4 μg/m^3^ Area sampling 14.9–27.3 μg/m^3^ [18]
Boiler installation	1976-2002 (25)	Installing boilers in homes	None specified	
Construction field				
		Manual labor on a construction site	Silica dust (quartz)	0.10 mg/m^3^ [26]
Apartment complex janitor	2003-2013 (10)	Gardening and janitorial duties including the night shift	None specified	

The working process at the factory is summarized as follows. Lumps of scrap metal were poured into a smelting furnace, melted, and then transported to a mold to be solidified. The solid mold was then sent to the heat treatment procedure area. The patient’s main duty was to transport the casting by hoisting it or using a crane to move it to the heating furnace, and then control the temperature. All of these processes including those at the smelting furnace were all performed in an area covering about 997.73 m^2^. Bunker-C oil was the fuel source for the heating furnace. The patient stated he had inhaled a large volume of smoke from the soot produced when controlling the temperature or turning the ignition on. There were no local ventilation systems, respiratory protectors, or partitions to isolate the shaking out and finishing processes; therefore, exposure to crystallized silica from adjacent areas is likely.

The patient typically worked two shifts each week and had a few days off monthly. In addition to working at the wheel manufacturing company, he also worked for 5 years as a laborer on construction sites. After he stopped working on construction sites, he installed boilers and worked general manual labor for approximately 25 years. Since 2003, he has been working as janitor in an apartment complex.

**Figure 1:**
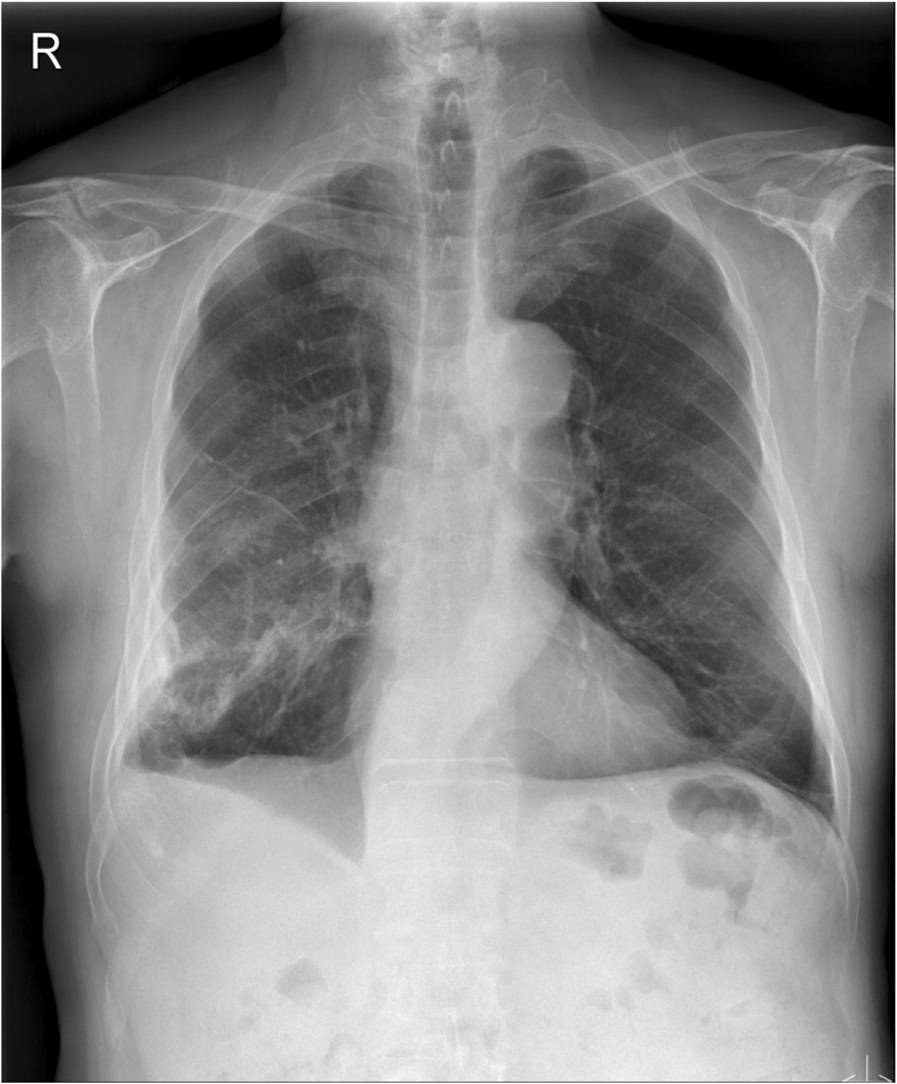
**Plain chest radiograph indicates lucent upper lobes in both lungs suggesting emphysema.** Evidence of lucent upper lobes in both lungs suggests emphysema. In addition, diffuse pleural thickenings with pleural calcifications present in the right middle and lower thorax. Linear atelectasis or fibrotic scars also present in the left lower lobe. Although the patient stated no history of asbestos exposure, asbestos exposure might have occurred while installing boilers.

**Figure 2:**
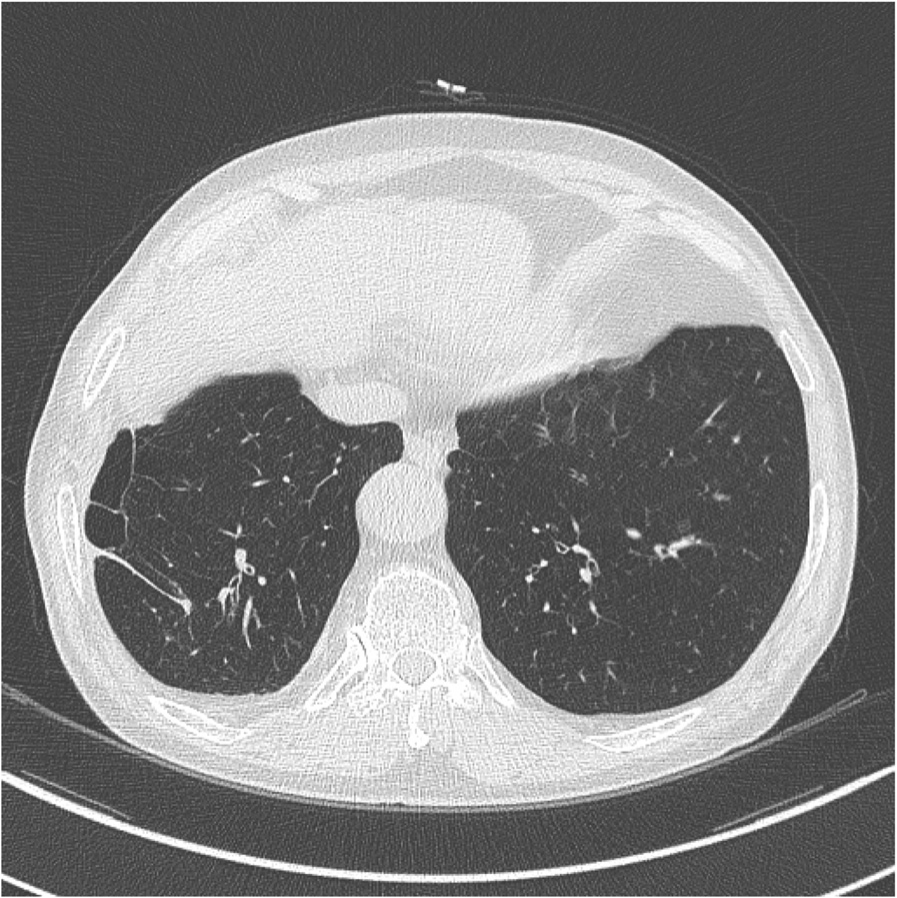
**Computed tomogram image indicating peripheral bullous changes in the right lower lobe.**

On May 21, 2012, a chest X-ray and CT scan revealed an evidence of hyperinflation with emphysematous changes in both upper lobes; he was diagnosed with emphysema. A month later (Figures 1, 2), a pulmonary function test was done that included a post-bronchodilator forced vital capacity (FVC), forced expiratory volume in one second (FEV_1_) and FEV_1_/FVC% that were 2.20 L (67% of the predicted value), 1.12 L (52% of the predicted value), and 51%, respectively, which all indicate moderate COPD.

## Conclusion

The patient had worked at wheel manufacturing plant mainly performing the heat treatment process for 10 years and also installed boilers for 25 years. The patient was exposed to the combustion of bunker C-oil, the fuel that heated the furnace, near the casting processing room without any respiratory protection or local ventilation systems. He was also exposed to crystallized silica from processes occurring in adjacent rooms. The factory housed six heating furnaces and a casting processing room in the same area, which were occasionally operated at the same time; we expect that the patient was exposed to a great volume of complete/incomplete combustion by-products of bunker C oil as well as crystallized silica. He was also likely to be exposed to silica while installing boilers and working on construction sites. Considering the various exposures to occupationally hazardous materials, we believe his COPD is related to the exposures in these work environments.

### Epidemiologic evidence on the relationship between COPD and occupational exposure

Several epidemiologic studies including population-based cohort studies have reported a relationship between COPD and occupational exposure [8-10]. According to the recent Swiss cohort with 4,267 non-asthmatic participants (from the general population, n = 9,561) who were collected at baseline in 1991 and followed-up until 2001–2003, the incidence rate of moderate (forced expiratory volume at timed intervals of 1 second, less than 80% of predicted) or severe COPD was 1.10 (95% CI: 1.02–1.19) among workers exposed to gas or fumes, 1.10 (95% CI: 1.01–1.20) among workers exposed to mineral dust, and 1.11 (95% CI: 1.04–1.17) among workers exposed to mixed dust [9]. Among non-smokers, the population attributable fraction for the association between occupational exposure and the range of the incidence of Global initiative for chronic obstructive lung disease (GOLD) stage II or higher was 43%–56% [9]. Moreover, an international ecological study predicted a 0.8% increase in the prevalence of occupational COPD with a 10% increase in its exposure prevalence [11]. This study also suggested that a 20% relative reduction in COPD can be accomplished with a 8.8% reduction in occupational exposure [11].

### Mechanisms of COPD by occupational exposure

Exposure to several kinds of dusts, metals, and endotoxins might result in chronic bronchitis with air obstruction according to two experimental animal models [12,13]. In human models, only alpha 1 anti-trypsin deficiency and an emphysema model confirmed these findings [14]. However, emphysema induced by exogenous exposure is likely to have different pathologic features from alpha 1 anti-trypsin deficiency (centrilobular vs. panacinar) [15]. Centrilobular emphysema due to occupationally hazardous materials might result from alveolar macrophages that can clear materials from the alveolars and bronchioles. Several experimental studies have suggested that a lack of macrophage metalloelastase might play a protective role against the development of emphysema [16,17]. Thus, alveolar macrophage exposure to occupationally hazardous materials might result in centrilobular emphysema.

### Possible risk factors of COPD from exposure estimations

Due to the limitations in estimating occupational exposures to evaluate the relationship between the work environment and COPD, an in-depth literature review on similarly exposed groups and/or workplaces was performed. In the 1990s, the geometric mean (GM) and geometric standard deviation (GSD) of the respiratory dust concentration according to personal and regional sampling at a casting workplace were 0.58 mg/m^3^ (GSD: 1.89; range: 0.16–3.10 mg/m^3^) and 0.40 mg/m^3^ (GSD: 2.11; range: 0.05–2.32 mg/m^3^), respectively, in South Korea [18]. The GM (GSD) of quartz concentrations in the personal and area sampling were 4.41 μg/m^3^ (3.05) and 14.88 μg/m^3^ (1.60), respectively, by X-ray fluorescence spectrometry [18]. The percentages of quartz by weight in the area and bulk sampling were 3.73% (1.51) and 11.56% (11.60) [18], respectively. In a similar study, the percent of quartz in a sample area ranged from 3.36% to 14.69% [19]. In the early 2000s, a study on the casting factory evaluated the concentration of quartz content in the air [20]. The GM concentration of quartz in 129 places in that foundry was 0.0273 mg/m^3^ (GSD: 3.9988; range: 0.0007–0.3757) and the proportion of the concentration that exceeded the threshold limit value and recommended exposure limit for quartz was 33% of the workplace [20]. The GM of the respiratory dust concentration in 129 personal samples was 1.2129 mg/m^3^ (GSD: 2.0772; range: 0.1524–7.0426) [20]. Phee et al. reported the exposure level of hazards including mineral dust at a foundry in 2002. The GM (GSD) of the total concentration of mineral dust were 1.10 (3.26), 0.88 (4.39), 1.26 (2.22), 1.53 (2.87), 1.07 (1.99), 1.75 (2.52), 2.32 (2.51) and 0.47 (4.57) and 0.90 mg/m^3^ in the melting pouring, molding, sand treatment, core making, shakeout, finishing, and miscellaneous areas, respectively [21].

According to a study on Polycyclic aromatic hydrocarbons (PAHs) and COPD, the concentration of PAHs was correlated with COPD and related mortality [22]. Our patient was exposed to the complete/incomplete combustion of bunker-C oil, and this exposure seems similar to that at the incineration plant. PAHs as a by-product of combustion might have been released at the incineration plant. A previous study reported that the concentration of PAHs in the incineration plant was 11,672.19 ng/Sm^3^ [23]. In addition, jet fuel combustion was found to result in a concentration of respirable particulate matter < 10 μm in a diameter at a peak level of 186 μg/m^3^ [8].

Our patient worked two jobs after quitting the wheel manufacturing plant. He worked as a boiler installer for 25 years and also worked on construction sites occasionally. One longitudinal cohort study on 196,329 Swedish construction workers showed high association between occupational exposure to vapors, gases, dusts, and fumes with COPD [24]. The attributable fraction of COPD with occupational exposure was 0.53 among never smoking workers [24]. Moreover, silica exposure is also prevalent in construction sites. According to a national survey from 1988 to 1994, 13% of construction worker had COPD, whereas the prevalence of COPD in the general population was estimated between 4% to 10% [25]. The mean exposure of respirable dust and quartz at construction sites in Germany was 0.88 mg/m^3^ (GSD: 4.23, range: 0.02–33.76) and 0.10 mg/m^3^ (GSD: 3.84, range: 0.01–1.36), respectively [26]. Moreover, cement dust, which the patient had been exposed to on construction sites, may have been an attributable exposure of occupational COPD.

### Exposure duration for diagnosing COPD

Obstructive patterns in pulmonary function tests have been found to be related to the duration of occupational exposure in a few studies. Angeles et al. reported subjects exposed to dust or fumes for longer than 15 years were likely to have lower pulmonary function than those exposed less than 15 years were. The mean difference in the FEV to FVC ratio was -1.7% (-3.3– -0.2) between those exposed longer than 15 years and those never exposed. Whereas, the FEV_1_ was -0.6 mL (-2.1– 0.8) between those exposed less than 15 years and those never exposed [27]. In a Swiss occupational cohort study, the minimum exposure durations for GOLD II or higher was 10–14 years, and that of GOLD I or higher was 6–10 years [9]. However, no standard of occupational exposure duration has been established and is still under discussion at the Korea Workers’ Compensation and Welfare Service.

### Smoking and COPD

Considering the patient’s 25 pack-year smoking history, it is difficult to distinguish between the effect of cigarette smoke and that of occupational exposures. However, previous studies emphasized that occupational exposure to dust, chemicals, and gases should be considered as an established risk factor for developing COPD independent of the effects of cigarette smoking [7,10,15]. One study found the unadjusted probability of developing COPD to be 0.10, 0.19, and 0.32 among those with only an occupational exposure, those with only a smoking exposure, and those with both exposures, respectively [10]. Several studies have emphasized the interaction between smoking and occupational exposures [10,28,29]. For example, one study showed that smoking and occupational factors together markedly increased the risk of developing COPD (OR 14.1; 95% CI: 9.33–21.20), whereas exposure to only smoking had a lower odds ratio than that of both exposures together (OR 6.71; 95% CI: 4.58-9.82) [28]. However, another study demonstrated that the combined effect of smoking with exposure to occupationally hazardous agents (biological dusts, mineral dusts, vapors, gases, other dusts or fumes) was likely an additive but not a multiplicative interaction [9]. A prospective study on smoking and lung function impairment in the UK found that the annual decline in FEV_1_ was 60 mL/year [30]. Therefore, the interactive effect between smoking and occupational exposure should be considered in work-related processes where the risk for occupational exposure is high.

### Limitations

Several limitations created difficulties in diagnosing occupational COPD in this patient. First, the previous workplace had been closed at patient presentation; therefore, monitoring of the work environment was not performed. However, we searched for similar results from similar work environments, and considered the patient’s work environment to be a poorer condition than those that we had found. The interaction between smoking and occupationally hazardous materials should also be considered. We attempted to investigate the combined effects of smoking and occupational exposure in our patient, but limitations in our ability to collect these data exist.

In conclusion, we described and considered the combined effects of a prolonged, complicated exposure to silica dust, gas, and fumes in the work place with smoking (25 pack-years) in a patient diagnosed with COPD. Further efforts to prevent the onset of COPD due to occupational exposures are needed.

## Consent

Written informed consent for publication of this case report including all radiologic findings was obtained from the patient.

## Abbreviations

COPD: Chronic obstructive pulmonary disease
FVC: Forced vital capacity
FEV_1_: Forced expiratory volume in one second
GM: Geometric mean
GOLD: Global initiative for chronic obstructive lung disease
GSD: Geometric standard deviation
PAHs: Polycyclic aromatic hydrocarbons
